# Individual Differences in Math Ability Determine Neurocognitive Processing of Arithmetic Complexity: A Combined fNIRS-EEG Study

**DOI:** 10.3389/fnhum.2019.00227

**Published:** 2019-07-03

**Authors:** Christina Artemenko, Mojtaba Soltanlou, Silke M. Bieck, Ann-Christine Ehlis, Thomas Dresler, Hans-Christoph Nuerk

**Affiliations:** ^1^LEAD Graduate School & Research Network, University of Tuebingen, Tuebingen, Germany; ^2^Department of Psychology, University of Tuebingen, Tuebingen, Germany; ^3^Leibniz-Institut für Wissensmedien, Tuebingen, Germany; ^4^Department of Psychiatry and Psychotherapy, University of Tuebingen, Tuebingen, Germany

**Keywords:** arithmetic complexity, math ability, individual differences, fNIRS, EEG

## Abstract

Some individuals experience more difficulties with math than others, in particular when arithmetic problems get more complex. Math ability, on one hand, and arithmetic complexity, on the other hand, seem to partly share neural underpinnings. This study addresses the question of whether this leads to an interaction of math ability and arithmetic complexity for multiplication and division on behavioral and neural levels. Previously screened individuals with high and low math ability solved multiplication and division problems in a written production paradigm while brain activation was assessed by combined functional near-infrared spectroscopy (fNIRS) and electroencephalography (EEG). Arithmetic complexity was manipulated by using single-digit operands for simple multiplication problems and operands between 2 and 19 for complex multiplication problems and the corresponding division problems. On the behavioral level, individuals with low math ability needed more time for calculation, especially for complex arithmetic. On the neural level, fNIRS results revealed that these individuals showed less activation in the left supramarginal gyrus (SMG), superior temporal gyrus (STG) and inferior frontal gyrus (IFG) than individuals with high math ability when solving complex compared to simple arithmetic. This reflects the greater use of arithmetic fact retrieval and also the more efficient processing of arithmetic complexity by individuals with high math ability. Oscillatory EEG analysis generally revealed theta and alpha desynchronization with increasing arithmetic complexity but showed no interaction with math ability. Because of the discovered interaction for behavior and brain activation, we conclude that the consideration of individual differences is essential when investigating the neurocognitive processing of arithmetic.

## Introduction

People differ in their math ability. Individual differences in math ability particularly matter when arithmetic problems get increasingly complex. Because with higher arithmetic complexity the difficulty level increases, individuals with low math ability, who might already struggle with simple arithmetic, experience even more difficulties when solving complex arithmetic problems (e.g., Artemenko et al., 2018b). The most frequently studied arithmetic operation in the context of arithmetic complexity and math ability is multiplication (e.g., Grabner et al., 2007; Soltanlou et al., 2018; for a review, see Zamarian et al., 2009). The aim of the current study is to replicate the findings regarding arithmetic complexity and math ability for multiplication and further explore the interaction of these factors in both multiplication and division, which is the inverse operation of multiplication and largely understudied. Studying division provides the chance to examine to what extent the findings from multiplication can be generalized.

The complexity of multiplication depends on several factors (for an overview, see Domahs et al., 2006), for example, the interference effect (De Visscher and Noël, 2014). One other important factor is the effect of problem size (Tiberghien et al., 2018), i.e., multiplication problems with numerically larger operands are more difficult to solve—as reflected by higher reaction times (RT) and error rates (ER)—than multiplication problems with smaller operands (Verguts and Fias, 2005). While this effect can be derived from the neighborhood consistency when smaller single-digit problems (e.g., 3 × 4) are compared to larger single-digit problems (e.g., 8 × 7; Domahs et al., 2006; for the interacting neighbors model see Verguts and Fias, 2005), the problem size effect might be more pronounced when comparing problems with single-digit operands only (e.g., 4 × 6) to problems with at least one two-digit operand (e.g., 16 × 4). The reason is that single-digit arithmetic facts from the multiplication table can mostly be retrieved from long-term memory (at least in adults), while two-digit multiplication rather necessitates procedural calculation strategies (Tronsky, 2005).

On the neural level, arithmetic fact retrieval, as the most used strategy for simple multiplication, is associated with left perisylvian language areas such as the superior temporal gyrus (STG) and the middle temporal gyrus (MTG), parietal areas in the left intraparietal lobule (IPL), i.e., angular gyrus (AG) and supramarginal gyrus (SMG; Dehaene and Cohen, 1997; Dehaene et al., 2003; Klein et al., 2016), and recently identified regions such as the hippocampus and the retrosplenial cortex (Cho et al., 2012; Klein et al., 2016, 2019). The most prominent region associated with arithmetic fact retrieval is the left AG, as it was found in several functional magnetic resonance imaging (fMRI) studies (Grabner et al., 2007, 2009a, 2013; Jost et al., 2009; De Visscher et al., 2015). This region is considered to be responsible for the automatic mapping between a multiplication problem and its solution. In electroencephalography (EEG) studies, arithmetic fact retrieval is assumed to be accompanied by higher theta synchronization (increase in theta power) especially in the left hemisphere (Earle et al., 1996; Harmony et al., 1999; Micheloyannis et al., 2005; De Smedt et al., 2009; Grabner and De Smedt, 2011; but see Moeller et al., 2010).

On the other hand, complex arithmetic, which is typically solved by applying procedural strategies, requires a rather widespread fronto-parietal network, as it was observed in fMRI studies (e.g., Gruber et al., 2001; Fehr et al., 2007; Grabner et al., 2009a). Thereby, higher activation with increasing complexity in multiplication was observed in frontal areas such as the left inferior frontal gyrus (IFG) and left middle frontal gyrus (MFG), reflecting domain-general cognitive task demands (e.g., maintenance of rule-based decomposed calculation steps), and the posterior parietal cortex, reflecting domain-specific numerical task demands (Gruber et al., 2001; Grabner et al., 2007; Jost et al., 2009; De Visscher et al., 2015; see also Menon et al., 2000; Zago et al., 2001; Tiberghien et al., 2018). In EEG studies, more alpha desynchronization (decrease in alpha power) was observed for complex, non-retrieved arithmetic problems (Harmony et al., 1999; Moeller et al., 2010; but see Micheloyannis et al., 2005). In general, alpha desynchronization is caused by increases in task complexity, attentional demands, cognitive load, and mental effort (Gevins et al., 1997; Pfurtscheller and Lopes da Silva, 1999; Antonenko et al., 2010). Thereby, lower alpha desynchronization (8–10 Hz) is considered to be task non-specific and topographically widespread at parieto-central sites reflecting attentional demands, while upper alpha desynchronization (10–13 Hz) is topographically more restricted to occipito-parietal sites, associated with processing of semantic information, and shows variations in response to the cognitive content of the task (Gevins et al., 1997; Klimesch, 1999; Pfurtscheller and Lopes da Silva, 1999). The differentiation within the alpha band seems to be important also in the context of arithmetic, since lower alpha was shown to be more sensitive to complexity and training effects than upper alpha (Grabner and De Smedt, 2011, 2012). Taken together, simple multiplication is usually solved by arithmetic fact retrieval and reflected by higher involvement of left-hemispheric language areas (e.g., AG) and theta synchronization, while complex multiplication is usually solved by procedural strategies and reflected by higher fronto-parietal activation (e.g., left IFG) and alpha desynchronization.

However, performance does not only depend on the complexity of the task, but also on the ability of the individual. Math ability generally improves with age, experience and training. Developmental studies have shown evidence for a shift from more frontal to more parietal activation with age so that arithmetic processing more and more relies on specific numerical magnitude processing and automatized fact retrieval (Rivera et al., 2005; Menon, 2010; Prado et al., 2014; Artemenko et al., 2018c; for a meta-analysis see Arsalidou et al., 2018; for a review, see Peters and De Smedt, 2018). Training studies suggest a shift within the parietal cortex from more effortful procedural processing in the IPS to fact retrieval in the left AG (Delazer et al., 2003, 2005; Grabner et al., 2009b; Ischebeck et al., 2009; for a review, see Zamarian et al., 2009), at least in adults (for children see e.g., Soltanlou et al., 2018). However, a recent fMRI study found the left AG not to be directly involved in fact retrieval after learning but rather a single part of the ventral stream areas in the parietal cortex (as is also the SMG) involved in bottom-up attentional processes emerging when faced with a learned multiplication problem (Bloechle et al., 2016). Nevertheless, the specific role of the left AG and the additional involvement of the MTG are further supported by studies considering individual differences in math ability for multiplication indicating less deactivation of these areas with higher math ability (Grabner et al., 2007; but see Rosenberg-Lee et al., 2011). Additionally, in division, the SPL was shown to be less activated in individuals with higher math ability (Rosenberg-Lee et al., 2011). In EEG studies, training in multiplication led to higher theta synchronization and less lower alpha desynchronization (Grabner and De Smedt, 2012; but see Earle, 1985). Less alpha desynchronization is in line with general ideas of good performance and practice effects, suggesting that for improvements in task performance fewer cortical resources are required for more skilled individuals compared to less skilled individuals (Gevins et al., 1997; see also Klimesch, 1999).

Taken together, simple multiplication facts are usually retrieved from semantic long-term memory and this process seems to be supported by left-hemispheric language areas, the AG in particular, and theta synchronization. With increasing arithmetic complexity, multiplication problems are solved by applying procedural strategies which necessitate increased fronto-parietal activation and lead to alpha desynchronization. Analogous to the effects of arithmetic complexity, individual differences in math ability were also found in the AG, theta and alpha frequency bands. Due to the similarity of the neural correlates for both arithmetic complexity and math ability, the question arises whether the processing of arithmetic complexity differs depending on math ability. In the case of addition and subtraction, an interaction was found in the behavioral measures of RT and ER, in the neural activation of the left IFG measured by functional near-infrared spectroscopy (fNIRS), and in the late components of event-related potentials (ERPs) at frontal sites (Artemenko et al., 2018b). For multiplication, a significant interaction was only found on the behavioral level as reflected by RT, but not on a neural level assessed by fMRI (Grabner et al., 2007; De Visscher et al., 2018). In regard to division, the interaction of arithmetic complexity and math ability has not been investigated so far. There seems to be a lack of evidence for multiplication and especially for division regarding this interaction in terms of neural activation and oscillation. To investigate this issue is crucial since both differences in arithmetic complexity as well as in math ability seem to point at the same neural mechanisms being different for arithmetic fact retrieval and procedural strategy use so that this needs to be empirically demonstrated.

This study aims at understanding the interaction of math ability with arithmetic complexity in multiplication and division on behavioral and neural levels. Since these factors on their own were already extensively studied in regards to multiplication, these findings might serve as a basis for interpreting the findings for division, which is the inverse arithmetic operation to multiplication (Campbell, 1997; LeFevre and Morris, 1999; Rosenberg-Lee et al., 2011; Huber et al., 2013), because division is usually not separately studied, but division stimuli are sometimes used in combination with other arithmetic operations (Gruber et al., 2001; Grabner et al., 2009a; Ischebeck et al., 2009; for exceptions, see Fehr et al., 2007; Rosenberg-Lee et al., 2011). In order to evaluate the correlates gained from neuroimaging as well as from neurophysiological measures, we will use combined fNIRS-EEG measurements. By using these methods in combination, it is further possible to use a (written) production paradigm (for verbal production paradigms see Gruber et al., 2001; Zago et al., 2001). This might allow for the measurement of arithmetic processing in individuals with high and low math performance in a more natural setting and to reduce additional decision and recognition processes occurring in verification and choice-reaction paradigms (Hinault and Lemaire, 2016). We hypothesize an interaction of math ability and arithmetic complexity on the behavioral level in terms of RT and ER as well as on the neural level in terms of activation differences in the left IFG and left AG and changes in theta synchronization and alpha desynchronization. Thereby, we expect an interaction in the left IFG also for multiplication and division because of similar findings for other arithmetic operations (Artemenko et al., 2018b). The hypothesized interactions in the left AG and in the theta and alpha frequency bands is based on the findings that these neural components are affected by both factors reflecting differences in arithmetic fact retrieval and procedural strategy use in multiplication so that an overadditive relation might be possible. The interaction should be investigated for both multiplication and division in order to determine to what extent the neural correlates are operation-specific and to what extent the network for fact retrieval, for example, generalizes across operations.

## Materials and Methods

### Participants

All 34 subjects (10 male; age: *M* = 24.5 years, *SD* = 5.3 years) who participated in this study were native German speakers, right-handed by means of scores between +40 and +100 in the Edinburgh-Handedness Inventory (Oldfield, 1971), showed no history of neurological or mental disorders, and had normal or corrected-to-normal vision. The subjects gave informed written consent and received monetary reimbursement for participation. This study was approved by the ethics committee of the Medical Faculty of the University of Tuebingen and was in line with the latest version of the Declaration of Helsinki.

Initially, 72 participants were recruited for an online screening of arithmetic abilities consisting of speeded addition, subtraction, multiplication and division. For each operation, the participants were asked to solve as many arithmetic problems as possible within the time limit of 2 min. After drop-out due to unavailability, the upper and lower tertile were chosen from the remaining 53 participants to constitute the groups for high and low math ability (*n* = 17 per group), respectively. As intended, the math ability groups differed significantly in the screening for multiplication (*t*_24.2_ = 6.39, *p* < 0.001; high: *M* = 26.18, *SD* = 6.18; low: *M* = 15.35, *SD* = 3.25) and division (*t*_21.9_ = 5.50, *p* < 0.001; high: *M* = 21.29, *SD* = 8.70; low: *M* = 8.62, *SD* = 3.80), and also in addition and subtraction (for details see Table 1 in Artemenko et al., 2018b). Furthermore, there were no group differences in age, sex, non-verbal intelligence, verbal short-term memory and visuo-spatial short-term memory (for details see Table 1 in Artemenko et al., 2018b); only verbal working memory capacity was significantly lower in individuals with low math ability (*t*_24.4_ = 2.42, *p* = 0.023).

### Materials

As arithmetic tasks, both multiplication and division tasks were employed including two complexity levels (50 arithmetic problems per condition). Simple multiplication problems included all possible combinations of two single-digit operands with a result in the range of 12–72 except for ties (see LeFevre et al., 2004). Complex multiplication problems consisted of one single-digit operand in the range of 2–9 and one two-digit operand in the range of 12–19, thereby excluding ties between the units. In each condition, the larger operand occurred with equal frequency at each position and the numerical size and the parity of the operands were matched. For division, the inverse multiplication problems were used (e.g., 9 × 8 → 72 ÷ 8).

For the assessment of strategy use in each task, four example problems for each complexity level were given in pseudorandomized order. The participants had to produce the solution of each problem and to report the used strategy by classifying it into one of the following categories: arithmetic fact retrieval, counting in steps, decomposition in units and decades, transformation to several calculation steps, rounding up or down, referring to related operations, using other/no strategies like guessing (see Campbell and Xue, 2001; Imbo and Vandierendonck, 2007). Before doing so, the participants received explanations and examples for these strategies.

### Procedure

The study was part of a larger project investigating the neurocognitive correlates of arithmetic processing (Artemenko et al., 2018a, b). During combined fNIRS-EEG measurements, computerized tasks (addition, subtraction, multiplication, division, number copying, letter copying) were administered in a light-attenuated room within two experimental sessions, with the order of tasks counterbalanced across subjects. In the current study, we focus on the behavioral, fNIRS, and EEG data for the multiplication and division tasks. After each task, strategy use was measured. In the end, intellectual capability, memory capacity, and motivation in math (not considered here) were assessed.

By means of Presentation software (NeuroBehavioral Systems, Inc., Berkeley, CA, USA), arithmetic problems were vertically centered and horizontally left-centered presented in white on a black background. In a written production paradigm, participants were asked to mentally calculate the arithmetic problem as quickly and accurately as possible and to write the solution behind the equal sign on the right side of the screen by using a touch pen on the touch screen (see Figure 1 in Artemenko et al., 2018b). Note that the written response was not visible to the participants in order to reinforce mental arithmetic and reduce movement artifacts during calculation. In an event-related design, a trial was terminated by the participants clicking on the gray box presented on the right or by reaching the time limit of 15 s and was followed by an inter-trial interval of 4–7 s (mean of 5.5 s, jittered in steps of 120 ms). Each arithmetic task had a randomized trial order for every participant and started with eight practice items.

### Data Acquisition

#### fNIRS

fNIRS data were collected with the help of an ETG-4000 Optical Topography System (Hitachi Medical Corporation, Tokyo, Japan). The light absorption was measured at two wavelengths (695 nm and 830 nm ± 20 nm) with a sampling rate of 10 Hz. In order to measure fNIRS and EEG data simultaneously, participants put on a combined fNIRS-EEG cap (Brain Products GmbH, Herrsching, Germany). fNIRS probesets embedded in this combined cap consisted of 22 channels for each hemisphere (overall 44 channels) with an inter-optode distance of 30 mm.

Due to individual head sizes of the participants, four different cap sizes were used (54, 56, 58 and 60 cm). Corresponding to the 10/20-system (Jasper, 1958), channels 14/40 of the probesets were placed at P3/P4 and oriented towards F3/F4 (for the location of the probeset and the channels see Figure 1 in Artemenko et al., 2018b). In order to determine the anatomical areas underneath the channels, mapping was provided by Ippeita Dan and Minako Uga based on a virtual registration method (Rorden and Brett, 2000; Singh et al., 2005; Tsuzuki et al., 2007) and labeled according to the automated anatomic labeling (AAL) atlas (Tzourio-Mazoyer et al., 2002).

#### EEG

EEG data were recorded using a 32-channel DC-amplifier and the software Brain Vision Recorder (Brain Products, Munich, Germany). For EEG, 21 scalp electrodes were used and embedded in the combined fNIRS-EEG cap according to the 10–5 system (Oostenveld and Praamstra, 2001) because of the fixed fNIRS optode positions (see Figure 1 in Artemenko et al., 2018b). The electrode was positioned at frontal (FP1, Fz, FP2, AFF7h instead of F7, AFF3 instead of F3, Fz, AFF4 instead of F4, AFF8h instead of F8), central (FCC3 instead of C3, Cz, FCC4 instead of C4), temporal (T7, T8), parietal (TPP7h instead of P7, CPP3 instead of P3, Pz, CPP4 instead of P4, TPP8h instead of P8), and occipital sites (O1, Oz, O2). To detect eye movement artifacts, electrooculography (EOG) was recorded by an additional electrode placed below the right eye. The ground electrode was placed at AFz and the online reference electrode at FCz. Electrode impedances were kept below 10 kΩ. The sampling rate was 1,000 Hz and an online bandpass filter of 0.1–100 Hz was applied to the signal.

### Data Analysis

#### Behavioral Data

The written responses were manually analyzed for correctness after visualization by the RON (ReadOut Numbers) program (Ploner, 2014). Only correctly solved trials were included in the response time (RT) and later in the fNIRS and EEG analyses. For RT analysis, RTs outside a range of 3 SD from the subject’s mean were repeatedly excluded in each task. For ER analysis, arcsine-square-root-transformed ER were used to approximate normal distribution (Winer et al., 1971). RT and ER were separately analyzed in 2 operation (multiplication, division) × 2 complexity (simple, complex) × 2 math ability (high, low) analysis of variances (ANOVAs) using SPSS (IBM SPSS Statistics). Three subjects were excluded from ER analysis because of technical problems in the recording of their written responses in the division task^1^. Strategy use was analyzed by a similar 2 × 2 × 2 ANOVA, after determining the amount of arithmetic fact retrieval use for each participant and condition.

#### fNIRS Data Analysis

Channel-specific differences in the light absorption and therefore concentration of both oxygenated (O_2_Hb) and deoxygenated hemoglobin (HHb) were obtained and used for further analysis. Analysis was carried out using customized Matlab (The MathWorks, Inc., Natick, MA, USA) scripts according to Artemenko et al. (2018b). In the first step, a bandpass filter (0.02–0.25 Hz) was applied, trials with uncorrectable artifacts were excluded (10% in multiplication; 10% in division) and noisy fNIRS channels were interpolated (3% in multiplication; 6% in division). In the second step, in order to reduce further noise arising from motion artifacts and other non-evoked systemic influences, correlation-based signal improvement (CBSI) was used, which is based on the negative correlation of O_2_Hb and HHb (Cui et al., 2010). Although the optimal correction approach for fNIRS is data-dependent, the CBSI method is considered to be a valid correction method (Brigadoi et al., 2014), especially in case of motion artifacts which are likely to occur in the current production paradigm. In the third step, a general linear model approach was applied with the hemodynamic response function having a peak at 9 s (see Artemenko et al., 2018b), to get the individual beta values for each channel, participant, and task. Statistical analyses were conducted following the procedure described in Artemenko et al. (2018b): one-sample *t*-tests were performed for the conditions (simple and complex) and a paired *t*-test was performed for the contrast of conditions (complex vs. simple) for each task and group [significance level of 0.05, Dubey/Armitage-Parmar (D/AP) corrected for multiple comparisons]. Additionally, independent two-sample *t*-tests were performed for the contrast [(complex vs. simple)_high_ − (complex vs. simple)_low_] to compare the complexity effects between the math ability groups (significance level of 0.05, D/AP corrected). The D/AP correction (Sankoh et al., 1997), a stepwise modified Bonferroni method which takes autocorrelations of data into account, was applied because it fits well to fNIRS data with its strong correlations between neighboring channels.

#### EEG Data Analysis

EEG data analysis was performed with Brainstorm (Tadel et al., 2011), which is documented and freely available for download online under the GNU general public license^2^. EEG data preprocessing included re-referencing to average reference and applying a bandpass filter of 0.1–40 Hz. Eye movement artifacts were detected by EOG and removed using signal space projections. Afterward, the continuous EEG data were epoched into 4 s for baseline (before stimulus onset), 4 s for simple trials, and 7 s for complex trials based on the mean RT across tasks and groups (after stimulus onset). For each condition and participant, power spectral density (PSD) was calculated for different frequency bands, as outlined above: theta (4–7 Hz), lower alpha (8–10 Hz), and upper alpha (10–13 Hz). The percentage values of event-related synchronization (ERS) or event-related desynchronization (ERD) were determined by ERS/ERD % = (PSD of activation − PSD of baseline)/PSD of baseline × 100 (Pfurtscheller and Lopes da Silva, 1999). ERS indicates a larger PSD for activation than baseline (positive difference) and ERD indicates a smaller PSD for activation then baseline (negative difference). Statistical analyses were performed in the same way as the statistical analyses of the fNIRS data (significance level of 0.05, Bonferroni corrected for multiple comparisons). The more conservative Bonferroni correction method was used for the EEG data (as compared to the fNIRS data) since EEG electrodes detect oscillations of the whole brain including subcortical structures and thus are not locally restricted to approximately 3 cm like the fNIRS channels. Because of technical problems no EEG data was recorded during the division task for one participant in the high math ability group.

## Results

### Behavioral Data

**Figure 1:**
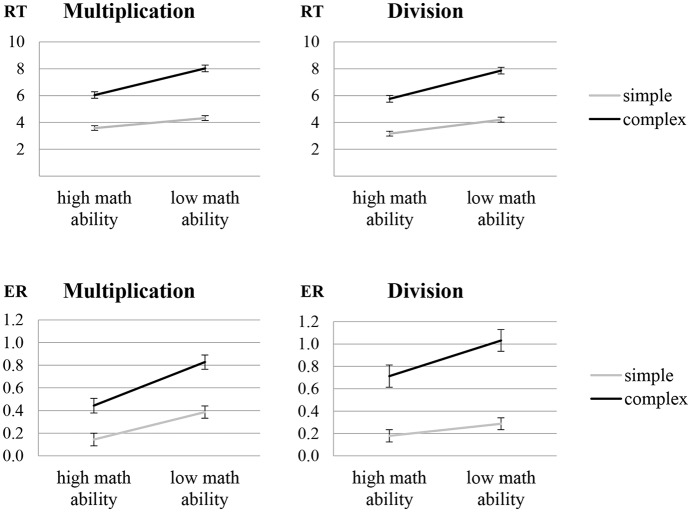
Behavioral data [response time (RT) in seconds and arcsine transformed error rates (ER)] for the multiplication and divisions tasks in particular showing an interaction of complexity and math ability. Error bars depict 1 *SE* of *M*.

In the analysis of RT, significant main effects were observed for complexity (*F*_1,32_ = 1341.99, *p* < 0.001, $\eta_{\text{p}}^{2}$ = 0.977) and math ability (*F*_1,32_ = 38.09, *p* < 0.001, $\eta_{\text{p}}^{2}$ = 0.543), indicating that participants were faster at solving simple rather than complex arithmetic problems and individuals with high math ability were faster than individuals with low math ability. Moreover, a significant interaction of complexity and math ability (*F*_1,32_ = 46.31, *p* < 0.001, $\eta_{\text{p}}^{2}$= 0.591; see Figure 1, upper panel) indicates that individuals with low math ability showed a larger complexity effect than individuals with high math ability (*t*_32_ = 7.34, *p* < 0.001). All other effects were not significant.

In the analysis of ER, significant main effects were observed for complexity (*F*_1,29_ = 241.31, *p* < 0.001, $\eta_{\text{p}}^{2}$ = 0.893), math ability (*F*_1,29_ = 14.06, *p* = 0.001, $\eta_{\text{p}}^{2}$ = 0.327) and operation (*F*_1,29_ = 7.01, *p* = 0.013, $\eta_{\text{p}}^{2}$ = 0.195), indicating higher ER for complex compared to simple arithmetic problems, for individuals with low compared to high math ability, and for division compared to multiplication. Furthermore, a significant interaction of complexity and math ability (*F*_1,29_ = 7.38, *p* = 0.011, $\eta_{\text{p}}^{2}$ = 0.203; see Figure 1, lower panel) indicates a larger complexity effect in individuals with low rather than high math ability (*t*_29_ = 2.61, *p* = 0.014), and a significant interaction of complexity and operation (*F*_1,29_ = 7.90, *p* = 0.009, $\eta_{\text{p}}^{2}$ = 0.214) indicates a larger arithmetic complexity effect in division rather than multiplication (*t*_30_ = 2.87, *p* = 0.008). All other effects were not significant.

The distribution of strategy use is displayed in Supplementary Table S1 in the Supplementary Material. The analysis of retrieval strategies revealed main effects of complexity (*F*_1,32_ = 575.11, *p* < 0.001, $\eta_{\text{p}}^{2}$ = 0.947) and math ability (*F*_1,32_ = 8.23, *p* = 0.007, $\eta_{\text{p}}^{2}$ = 0.205), indicating that the participants solved simple arithmetic more often by arithmetic fact retrieval than complex arithmetic and individuals with high math ability relied more often on arithmetic fact retrieval than individuals with low math ability. All other effects were not significant so that, for example, no significant difference in strategy use was found between multiplication and division.

### fNIRS Data

**Figure 2:**
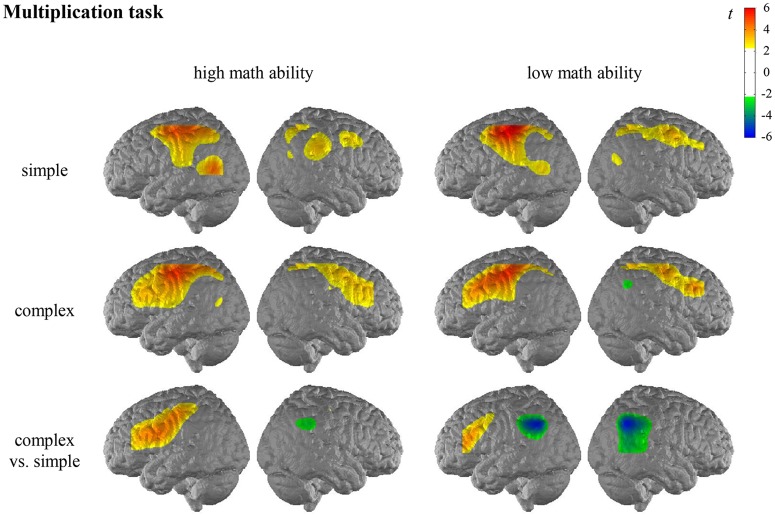
*T* maps for the functional near-infrared spectroscopy (fNIRS) data depicting neural activation during the multiplication task for simple arithmetic problems, complex arithmetic problems and the contrast (complex vs. simple) for individuals with high math ability and low math ability. The colors indicate activation (yellow-red) and deactivation (green-blue), respectively.

**Figure 3:**
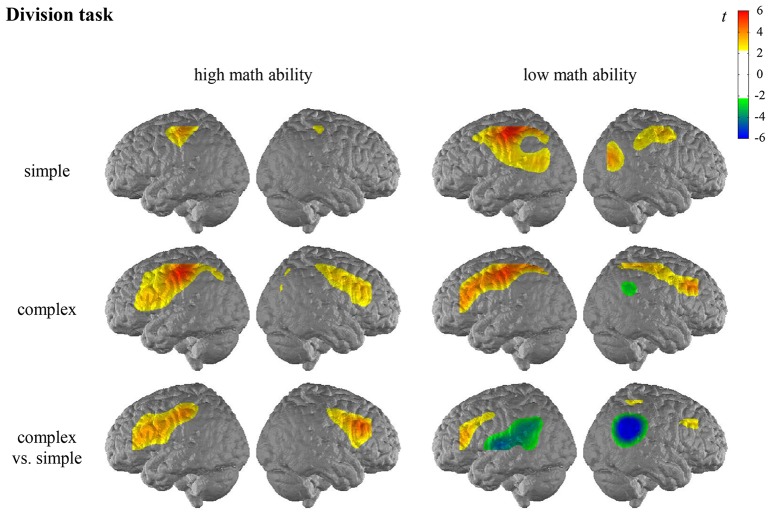
*T* maps for the fNIRS data depicting neural activation during the division task for simple arithmetic problems, complex arithmetic problems and the contrast (complex vs. simple) for individuals with high math ability and low math ability. The colors indicate activation (yellow-red) and deactivation (green-blue), respectively.

For the general activation patterns for individuals with high/low math ability in simple/complex multiplication/division, see Figures 2, 3 and Supplementary Tables S2, S3 in the Supplementary Material.

In the multiplication task, the contrast for individuals with high math ability revealed significantly higher activation of the left IFG and the left postcentral gyrus in complex compared to simple multiplication. The contrast for individuals with low math ability revealed significantly higher activation of the left IFG and significantly lower activation of the bilateral AG, bilateral SMG, and the right MTG in complex compared to simple multiplication.

**Figure 4:**
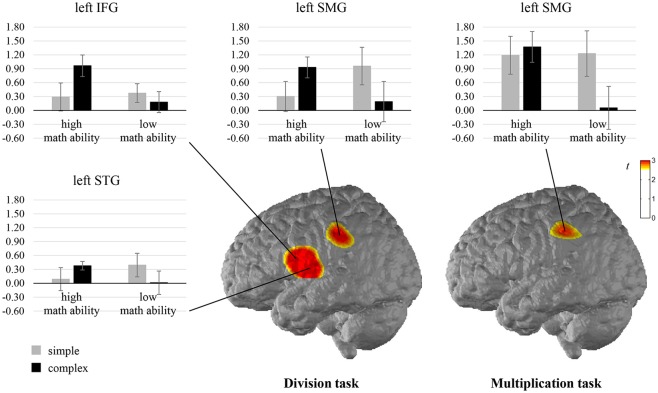
*T* maps for the fNIRS data depicting differences in neural activation between individuals with high and low math ability in the complexity effect in the multiplication and division tasks. Abbreviations: IFG, inferior frontal gyrus; STG, superior temporal gyrus; SMG, supramarginal gyrus.

When comparing the complexity effect between individuals with high and low math ability, significant differences were observed for the left SMG in the multiplication task, indicating that only individuals with low math ability show less activation in the left SMG for complex compared to simple multiplication (see Figure 4, Supplementary Table S2 in the Supplementary Material). All other comparisons were not significant.

In the division task, the contrast for individuals with high math ability revealed significantly higher activation of the bilateral IFG, the right MFG, and the left postcentral gyrus when comparing complex and simple arithmetic problems. The contrast for individuals with low math ability revealed significantly higher activation of the left IFG as well as significantly lower activation of the bilateral AG and the bilateral SMG, the left STG and the right MTG in complex compared to simple division.

The comparison of the complexity effect between individuals with high and low math ability revealed significant differences for the left SMG, the left IFG, and the left STG in the division task (see Figure 4, Supplementary Table S3 in the Supplementary Material). This indicates that individuals with low math ability show less activation in the left SMG for complex than simple division, and individuals with high math ability show more activation. Furthermore, only individuals with high math ability show higher activation in the left IFG for complex rather than simple division, and only individuals with low math ability show less activation in the left STG for complex rather than simple division. All other comparisons were not significant.

### EEG Data

For the general theta/alpha synchronization/desynchronization patterns for individuals with high/low math ability in simple/complex multiplication/division see Supplementary Tables S4, S5 in the Supplementary Material.

**Figure 5:**
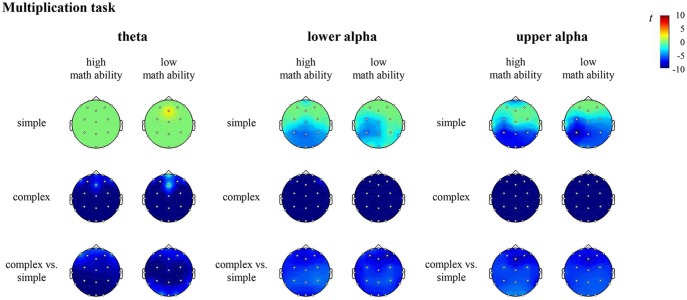
*T* maps for the electroencephalography (EEG) data depicting theta, lower and upper alpha (de)synchronization during the multiplication task for simple arithmetic problems, complex arithmetic problems and the contrast (complex vs. simple) for individuals with high math ability and low math ability. The colors indicate synchronization (yellow-red) and desynchronization (light-dark blue), respectively.

In the multiplication task, the contrast for individuals with high math ability revealed significantly higher desynchronization of theta, lower alpha and upper alpha at all sites (all channels) in complex compared to simple multiplication. The contrast for individuals with low math ability revealed significantly higher desynchronization of theta, lower alpha and upper alpha at all sites (all channels) in complex compared to simple multiplication (see Figure 5, Supplementary Table S4 in the Supplementary Material).

**Figure 6:**
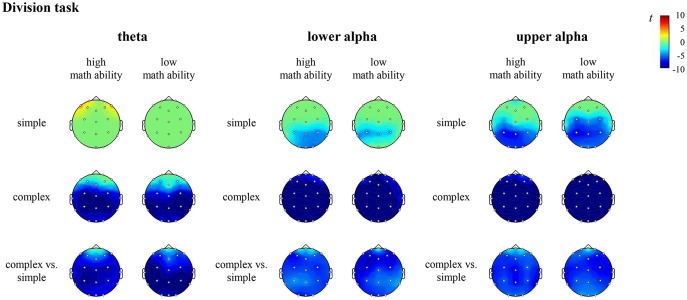
*T* maps for the EEG data depicting theta, lower and upper alpha (de)synchronization during the division task for simple arithmetic problems, complex arithmetic problems and the contrast (complex vs. simple) for individuals with high math ability and low math ability. The colors indicate synchronization (yellow-red) and desynchronization (light-dark blue), respectively.

In the division task, the contrast for individuals with high math ability revealed significantly higher desynchronization of theta at all sites (all channels except FP1, FPz, FP2), lower alpha and upper alpha at all sites (all channels except FP1, FP2) in complex compared to simple division. The contrast for individuals with low math ability revealed significantly higher desynchronization of theta, lower alpha and upper alpha at all sites (all channels except FPz) in complex compared to simple division (see Figure 6, Supplementary Table S5 in the Supplementary Material).

For both multiplication and division, the comparison of the complexity effect between individuals with high and low math ability revealed no significant differences. All other comparisons were also not significant.

## Discussion

In this study, the interaction of math ability with arithmetic complexity was investigated for multiplication and division by means of combined fNIRS-EEG measurements. With increasing arithmetic complexity, we observed less activation in the AG, SMG and right MTG and higher activation, especially in the left IFG. Importantly, left-hemispheric activation in the SMG for multiplication and in the SMG, STG and IFG for division further differed between individuals with high and low math ability, i.e., individuals with high math ability used these regions more for processing arithmetic complexity. This suggests an interaction of math ability with arithmetic complexity for multiplication and in particular for division. We found the interaction behaviorally in RT and ER as expected, in activation differences in the left IFG as expected and in the left SMG instead of the left AG, but not as changes in theta synchronization and alpha desynchronization. For the EEG data, we only found that the processing of arithmetic complexity leads to theta desynchronization and lower and upper alpha desynchronization, independent of the arithmetic operation.

In contrast to complex arithmetic problems, simple arithmetic problems are mostly solved by arithmetic fact retrieval and therefore in this study were found to be accompanied by more activation in areas responsible for verbally mediated arithmetic fact retrieval, i.e., AG, SMG and right MTG. This result fits with current models of number processing with the representation of verbal codes in perisylvian language areas (like the MTG and STG) and in the IPL (consisting of AG and SMG; Dehaene and Cohen, 1997; Dehaene et al., 2003; Klein et al., 2016), although the observed activation was not restricted to the left hemisphere. Further, this activation pattern is in line with previous studies on arithmetic complexity of multiplication (Grabner et al., 2007, 2009a, 2013; Jost et al., 2009; De Visscher et al., 2015), which usually emphasize the role of the left AG in mapping the multiplication problem to its solution. All of these results also hold for division, which is supported by a high correlation between simple multiplication and division in particular (Huber et al., 2013). This means that arithmetic fact retrieval in division is also associated with activation in the AG, SMG and right MTG. Consequently, our data suggest that the neural correlates of retrieval for multiplication facts also apply to retrieval for division facts and therefore should be generalized to arithmetic fact retrieval *per se*. This means that the mapping of an arithmetic problem to its result, mainly supported by the IPL, seems to be rather independent of the arithmetic operation.

Some of these areas in the IPL and temporal lobe, which were found to be associated with fact retrieval, were further shaped by math ability. Such individual differences for the arithmetic complexity effect could be shown for the left SMG in both multiplication and division, and for the left STG in division only. For the left SMG, only individuals with low math ability showed higher activation during simple rather than complex arithmetic, reflecting the use of fact retrieval almost only for simple problems. In contrast, individuals with high math ability generally used more retrieval strategies (i.e., also during complex arithmetic) than individuals with low math ability and therefore did not show similar activation differences in the left SMG. The role of the left SMG in arithmetic fact retrieval and its variation due to math ability is supported by previous research (Price et al., 2013). Furthermore, the left SMG is generally associated with symbolic number processing (Ansari, 2008) and phonological processing (Paulesu et al., 1993). Therefore, the left SMG might also support the mapping of an arithmetic problem to its solution similar to the left AG, which was typically shown to be affected by individual differences (Grabner et al., 2007). However, an interaction of arithmetic complexity and math ability could not be shown for the left AG (Grabner et al., 2007), instead, our results show this interaction for the left SMG. Although the study by Grabner et al. (2007) was quite similar to the current study, it differed in the neuroimaging method (fMRI vs. fNIRS) and paradigm (verification vs. production). In addition, activation in the left STG regarding arithmetic complexity also showed modulations due to math ability, i.e., the left STG was more activated in simple than complex division in individuals with low math ability only. This area is associated with the verbal representation of number processing (Klein et al., 2016) so that individuals with low math ability, who did not rely on fact retrieval for complex division, did show a higher activation difference in this area between solving simple and complex problems. In sum, the activation in the SMG and left STG varied for the arithmetic complexity effect depending on math ability, resembling the higher use of arithmetic fact retrieval in individuals with high math ability even for complex arithmetic. This interaction was shown here for the first time and seems to reflect interindividual differences in fact retrieval in the SMG. For division, this interaction additionally holds for the left STG, indicating more widespread activation differences for inverse operations (see also Artemenko et al., 2018b).

The areas discussed so far belong to the network for arithmetic fact retrieval, but with increased arithmetic complexity further effects were found. In the current study, arithmetic complexity was shown to be associated with higher activation in the frontal cortex, especially in the left IFG. Frontal activation reflects general increased task demands associated with more complex problems which are usually solved by procedural strategies (e.g., Grabner et al., 2007). The left IFG, in particular, was previously found to be more strongly activated for multi-digit than single-digit multiplication and division (Gruber et al., 2001; Grabner et al., 2007). Since the left IFG is related to verbal processing and working memory (for a meta-analysis see Liakakis et al., 2011), its increased activation during complex arithmetic might reflect the control, verbal rehearsal and maintenance of decomposed calculation steps during the application of rules (e.g., Gruber et al., 2001; Jost et al., 2009). Similar to the neural correlates of arithmetic fact retrieval, the correlates of arithmetic complexity in the left IFG, seem to also be generalizable and operation-independent because they were found for both multiplication and division.

The activation increase in the left IFG due to arithmetic complexity in the division task depended further on math ability. This interaction can be traced back to the finding that arithmetic complexity was associated with a more widespread frontal activation in the IFG for individuals with high rather than low math ability. This greater involvement of the left IFG for decomposing the complex arithmetic problem and keeping track of the calculation steps probably reflects the fact that individuals with high math ability more effectively process complex arithmetic, which is mostly solved by procedural strategies. This interpretation is corroborated by the findings that individuals with high math ability also have a smaller behavioral complexity effect and a larger verbal working memory capacity compared to individuals with low math ability. This is because the activation difference was observed in a domain-general cognitive processing area, which is associated with executive functions and working memory supporting arithmetic processing (Dehaene et al., 2003). The higher IFG activation in individuals with high math ability might, therefore, represent the higher verbal working memory capacity of these individuals and therefore lead to better performance in complex arithmetic. Individual differences in the activation of the left IFG were already shown for effects of arithmetic complexity in terms of the carry effect in addition (Artemenko et al., 2018b) and the interference effect in multiplication (De Visscher et al., 2018), although problem size was controlled for in these studies, but not for effects of problem size in the range of single-digit multiplication (De Visscher et al., 2018). In our study, division revealed these differences that were due to math ability, while we compared division as the inverse of multiplication with one two-digit operand with the inverse of single-digit multiplication. The reason might be the higher difficulty increase in division than multiplication as reflected by ER and the higher demand of decomposing complex division problems. Altogether, individuals with high math ability make use of the left IFG and thus more efficiently process the calculation steps needed for complex division.

Regarding the EEG results, increased arithmetic complexity leads to higher desynchronization in theta, lower and upper alpha, but was not modulated by math ability. In line with the literature, lower alpha desynchronization reflects possibly domain-general increased task demands elicited by more complex problems requiring procedural strategy use (De Smedt et al., 2009; Grabner and De Smedt, 2012; see also Antonenko et al., 2010). Therefore, alpha desynchronization corroborates the fNIRS result of increased frontal activation and provides evidence for operation-independent increased difficulty for processing arithmetic complexity. Contrarily, the interpretation of the result for theta is not that clear. On the one hand, the finding of higher theta synchronization for simple compared to complex arithmetic is line with studies considering theta synchronization to reflect arithmetic fact retrieval (De Smedt et al., 2009; Grabner and De Smedt, 2012). However, while this contrast is in line with these studies, the observed theta desynchronization for complex arithmetic was not reported before and might be a sign of differences in the baseline between the current and previous studies (e.g., due to timing). Moreover, theta synchronization was generally reported to increase with domain-general factors like task difficulty, demands of focused attention and working memory load (Gevins et al., 1997; for a review, see Klimesch, 1999)—contradicting our result of a decrease of theta synchronization for more complex problems. While we cannot resolve this issue with our current data (because of the restricted quality of EEG data due to the production paradigm, see Artemenko et al., 2018b), we recommend for further research to consider methodological details (e.g., regarding baseline and timing), different frequency bands (e.g., beta and delta), topography (e.g., high-resolution EEG) and more advanced analysis methods (Micheloyannis et al., 2005; e.g., coherence analyses, Varela et al., 2001; Molnár et al., 2009).

In conclusion, arithmetic fact retrieval involves the IPL (AG and SMG) and theta synchronization, and arithmetic complexity leads to left frontal activation (IFG) and alpha desynchronization. However, brain activation for the arithmetic complexity effect differed between individuals with high and low math ability: on the one hand, individual differences due to math ability affected processing of arithmetic facts in the left SMG for both multiplication and division. This reflects the greater use of arithmetic fact retrieval additionally for complex problems in individuals with high math ability. On the other hand, math ability influenced arithmetic complexity processing of division in the left IFG. This suggests that individuals with high math ability are more capable of decomposing complex division problems and keeping track of intermediate results. This study, therefore, replicates previous findings for multiplication and extends them to the understudied operation of division. In general, this might suggest that division partly involves similar processes such as a common representation of arithmetic fact retrieval in the IPL but is more affected by math ability for processing arithmetic complexity, like decomposing complex problems.

## Data Availability

The datasets generated for this study are available on request to the corresponding author.

## Ethics Statement

This study was approved by the ethics committee of the Medical Faculty of the University of Tuebingen. All subjects gave written informed consent in accordance with the Declaration of Helsinki.

## Author Contributions

CA, MS, A-CE, TD and H-CN designed and conceptualized the study. CA and MS collected data. CA and SB analyzed data. CA wrote the first draft of the manuscript. All authors reviewed the manuscript.

## Conflict of Interest Statement

The authors declare that the research was conducted in the absence of any commercial or financial relationships that could be construed as a potential conflict of interest.

## Footnotes

^1^Note that the RT, fNIRS and EEG data of these subjects were used in further analyses without excluding trials which were not correctly solved because of the missing ER data in the division task for these three subjects.

^2^http://neuroimage.usc.edu/brainstorm
