# Supplementary material for: Individual Differences in Math Ability Determine Neurocognitive Processing of Arithmetic Complexity: A Combined fNIRS-EEG Study
Source: Front Hum Neurosci. 2019 Jul 3;13:227. doi: 10.3389/fnhum.2019.00227 (PMC6616314; doi:10.3389/fnhum.2019.00227)
Supplement: Supplementary file 1 [file Data_Sheet_1.PDF]

Supplementary Material

Table S1: Strategy use during multiplication and division in individuals with high and low math ability.

task	complexity	math ability	retrieve	count	decomp	transform	round	operation
multiplication	simple	high	89.71%	5.88%			2.94%	1.47%
		low	82.35%	14.71%			1.47%	1.47%
	complex	high	16.42%	10.45%	35.82%	25.37%	8.96%	2.99%
		low	2.94%	11.76%	54.41%	25.00%	4.41%	1.47%
division	simple	high	95.52%	2.99%			1.49%	
		low	82.35%	8.82%		1.47%	1.47%	5.88%
	complex	high	13.33%	6.67%	15.00%	33.33%	16.67%	15.00%
		low	1.56%	29.69%	15.63%	39.06%	7.81%	6.25%

Categories of strategy use: arithmetic fact retrieval (retrieve), counting in steps (count), decomposition in units and decades (decomp), transformation to several calculation steps (transform), rounding up or down (round), referring to related operations (operation). Other strategies or missing values were excluded.

Table S2: Neural activation assessed by fNIRS during multiplication in individuals with high and low math ability.

area	L ch	*t*	*p*	R ch	*t*	*p*
high math ability, simple						
SPL	L 19	3.62	.000	R 44	3.02	.008
SMG	L 7	3.01	.008	R 29	2.58	.020
	L 11	2.90	.010	R 34	3.04	.008
	L 15	3.68	.002			
MTG	L 1	4.41	.000			
MFG				R 37	2.92	.010
PreCG	L 21	4.61	.000			
PostCG	L 16	4.17	.001			
	L 20	4.52	.000			
high math ability, complex						
SPL	L 19	3.94	.001	R 44	2.95	.009
SMG	L 7	2.72	.015			
	L 15	2.93	.010			
IFG	L 8	3.02	.008	R 32	3.19	.006
	L 13	3.39	.004			
MFG	L 17	3.19	.006	R 36	2.81	.013
				R 37	3.17	.006
PreCG	L 21	4.78	.000	R 42	2.98	.009
PostCG	L 12	3.71	.002			
	L 16	4.83	.001			
	L 20	4.27	.001			
high math ability, complex vs. simple						
IFG	L 8	3.95	.001			
	L 9	2.83	.012			
	L 13	3.86	.001			
PostCG	L 12	3.94	.001			
	L 16	3.92	.001			
low math ability, simple						
SPL	L 19	2.91	.010	R 44	3.01	.008
SMG	L 7	3.07	.007			
	L 11	2.50	.024			
MTG	L 1	2.81	.013			
MFG	L 17	2.81	.013	R 36	2.45	.026
				R 37	2.83	.012
PreCG	L 21	5.50	.000	R 42	3.31	.004
PostCG	L 12	3.20	.006	R 38	2.49	.024
	L 16	5.14	.000			
	L 20	5.28	.000			
low math ability, complex						
SPL	L 19	3.15	.006	R 44	3.02	.008
AG				R 35	-2.62	.018
SMG	L 7	2.62	.018			
IFG	L 13	3.98	.001	R 32	3.80	.002
MFG	L 17	4.51	.000	R 36	2.94	.010
				R 37	2.60	.019
PreCG	L 21	4.30	.001	R 42	3.51	.003
PostCG	L 12	3.78	.002	R 38	2.52	.023
	L 16	4.70	.001	R 43	2.73	.015
	L 20	4.29	.001			
low math ability, complex vs. simple						
AG	L 10	-4.98	.000	R 35	-5.41	.000
SMG	L 6	-3.89	.001	R 30	-4.04	.001
	L 11	-4.17	.001	R 34	-3.20	.006
MTG				R 26	-3.28	.005
IFG	L 9	3.66	.002			
	L 13	3.53	.000			
high vs. low math ability, complex vs. simple						
SMG	L 11	2.73	.010			

Note that only significant channels ($p < .05$, D/AP corrected) on the left (L ch) and right (R ch) hemispheres are given.

Table S3: Neural activation assessed by fNIRS during division in individuals with high and low math ability.

area	L ch	*t*	*p*	R ch	*t*	*p*
high math ability, simple						
PreCG	L 21	2.54	.022			
PostCG	L 16	3.09	.007			
	L 20	3.28	.005			
high math ability, complex						
SPL	L 19	2.54	.022			
AG	L 14	2.63	.018			
IFG	L 8	3.14	.006	R 32	3.34	.004
	L 13	2.99	.009			
MFG	L 17	2.56	.021	R 36	2.50	.024
				R 37	2.89	.011
PreCG	L 21	3.69	.002			
PostCG	L 12	3.59	.002			
	L 16	5.30	.000			
	L 20	4.34	.001			
high math ability, complex vs. simple						
IFG	L 8	3.35	.004	R 32	4.75	.000
	L 9	3.26	.005			
	L 13	3.54	.003			
MFG				R 36	3.10	.007
				R 37	2.93	.010
PostCG	L 12	3.37	.004			
	L 16	3.97	.001			
low math ability, simple						
SPL	L 19	4.07	.001			
AG				R 31	3.81	.002
SMG	L 6	2.52	.023	R 34	2.69	.016
	L 7	2.93	.010			
MTG	L 1	2.74	.014			
MFG	L 17	2.52	.023			
PreCG	L 21	4.07	.001	R 42	3.25	.005
PostCG	L 12	2.80	.013	R 38	2.53	.022
	L 16	4.98	.000			
	L 20	5.28	.000			
low math ability, complex						
SPL	L 19	4.08	.001	R 44	2.79	.013
IFG	L 9	3.55	.003			
	L 13	3.98	.001	R 32	3.74	.002
MFG	L 17	3.95	.001	R 36	3.46	.002
	L 18	2.49	.024	R 37	2.26	.018
PreCG	L 21	3.83	.001	R 42	2.81	.013
PostCG	L 12	3.19	.006	R 43	3.29	.005
	L 16	4.70	.000			
	L 20	4.02	.001			
low math ability, complex vs. simple						
AG	L 10	-3.26	.005	R 35	-4.41	.000
SMG	L 6	-3.95	.001	R 30	-8.45	.000
	L 11	-2.84	.012	R 34	-3.53	.000
STG	L 2	-3.74	.002			
	L 3	-4.63	.000			
MTG				R 26	-3.13	.007
IFG	L 9	3.33	.004			
high vs. low math ability, complex vs. simple						
SMG	L 11	3.11	.004			
STG	L 3	3.36	.003			
IFG	L 8	3.53	.001			

Note that only significant channels ($p < .05$, D/AP corrected) on the left (L ch) and right (R ch) hemispheres are given. Abbreviations: SPL – superior parietal lobule, AG – angular gyrus, SMG – supramarginal gyrus, STG – superior temporal gyrus, MTG – middle temporal gyrus, IFG – inferior frontal gyrus, MFG – middle frontal gyrus, PreCG – precentral gyrus, PostCG – postcentral gyrus.

Table S4: Theta, lower and upper alpha ERS/ERD during multiplication in individuals with high and low math ability.

site	theta	*t*	*p*	lower alpha	*t*	*p*	upper alpha	*t*	*p*
high math ability, simple									
frontal	Fp1			Fp1			Fp1	-4.98	< .001
	FPz			FPz	-4.38	< .001	FPz	-5.90	< .001
	Fp2			Fp2			Fp2	-4.52	< .001
	AFF7h			AFF7h			AFF7h		
	AFF3			AFF3			AFF3		
	Fz			Fz			Fz		
	AFF4			AFF4			AFF4		
	AFF8h			AFF8h			AFF8h		
temporal	T7			T7			T7		
central	FCC3			FCC3			FCC3	-4.79	< .001
	Cz			Cz	-4.07	< .001	Cz		
	FCC4			FCC4	-1.82		FCC4		
temporal	T8			T8	-0.97		T8		
parietal	TPP7h			TPP7h	-4.25	< .001	TPP7h	-7.02	< .001
	CPP3			CPP3	-5.76	< .001	CPP3	-9.04	< .001
	Pz			Pz	-5.75	< .001	Pz	-7.71	< .001
	CPP4			CPP4	-4.63	< .001	CPP4	-6.32	< .001
	TPP8h			TPP8h	-4.49	< .001	TPP8h	-7.05	< .001
occipital	O1			O1	-5.04	< .001	O1	-7.39	< .001
	Oz			Oz	-4.92	< .001	Oz	-7.14	< .001
	O2			O2	-5.54	< .001	O2	-8.13	< .001
high math ability, complex									
frontal	Fp1	-9.41	< .001	Fp1	-10.94	< .001	Fp1	-18.31	< .001
	FPz	-5.93	< .001	FPz	-13.23	< .001	FPz	-15.90	< .001
	Fp2	-8.12	< .001	Fp2	-10.59	< .001	Fp2	-19.45	< .001
	AFF7h	-4.29	< .001	AFF7h	-8.33	< .001	AFF7h	-10.29	< .001
	AFF3	-11.46	< .001	AFF3	-12.01	< .001	AFF3	-17.69	< .001
	Fz	-4.82	< .001	Fz	-12.37	< .001	Fz	-17.51	< .001
	AFF4	-10.25	< .001	AFF4	-9.92	< .001	AFF4	-13.51	< .001
	AFF8h	-5.00	< .001	AFF8h	-6.17	< .001	AFF8h	-9.58	< .001
temporal	T7	-12.34	< .001	T7	-10.87	< .001	T7	-12.52	< .001
central	FCC3	-11.98	< .001	FCC3	-11.42	< .001	FCC3	-17.77	< .001
	Cz	-9.23	< .001	Cz	-11.27	< .001	Cz	-16.89	< .001
	FCC4	-14.02	< .001	FCC4	-11.27	< .001	FCC4	-13.57	< .001
temporal	T8	-11.74	< .001	T8	-9.17	< .001	T8	-14.72	< .001
parietal	TPP7h	-11.14	< .001	TPP7h	-13.98	< .001	TPP7h	-23.23	< .001
	CPP3	-9.32	< .001	CPP3	-13.03	< .001	CPP3	-25.18	< .001
	Pz	-10.99	< .001	Pz	-12.98	< .001	Pz	-27.93	< .001
	CPP4	-11.28	< .001	CPP4	-14.57	< .001	CPP4	-22.14	< .001
	TPP8h	-11.62	< .001	TPP8h	-15.74	< .001	TPP8h	-25.01	< .001
occipital	O1	-10.88	< .001	O1	-13.30	< .001	O1	-20.08	< .001
	Oz	-10.94	< .001	Oz	-13.12	< .001	Oz	-18.90	< .001
	O2	-10.23	< .001	O2	-13.36	< .001	O2	-20.16	< .001
high math ability, complex vs. simple									
frontal	Fp1	-6.30	< .001	Fp1	-7.32	< .001	Fp1	-8.71	< .001
	FPz	-5.83	< .001	FPz	-8.70	< .001	FPz	-7.44	< .001
	Fp2	-5.12	< .001	Fp2	-5.44	< .001	Fp2	-6.00	< .001
	AFF7h	-4.10	< .001	AFF7h	-6.24	< .001	AFF7h	-6.21	< .001
	AFF3	-8.01	< .001	AFF3	-8.60	< .001	AFF3	-6.85	< .001
	Fz	-8.77	< .001	Fz	-8.64	< .001	Fz	-10.47	< .001
	AFF4	-10.25	< .001	AFF4	-8.10	< .001	AFF4	-9.02	< .001
	AFF8h	-4.43	< .001	AFF8h	-7.84	< .001	AFF8h	-8.92	< .001
temporal	T7	-10.22	< .001	T7	-7.50	< .001	T7	-6.34	< .001
central	FCC3	-9.24	< .001	FCC3	-6.78	< .001	FCC3	-5.65	< .001
	Cz	-8.88	< .001	Cz	-5.93	< .001	Cz	-6.19	< .001
	FCC4	-9.21	< .001	FCC4	-5.75	< .001	FCC4	-5.65	< .001
temporal	T8	-8.92	< .001	T8	-5.45	< .001	T8	-6.93	< .001
parietal	TPP7h	-10.83	< .001	TPP7h	-6.10	< .001	TPP7h	-6.65	< .001
	CPP3	-10.54	< .001	CPP3	-5.54	< .001	CPP3	-6.60	< .001

	Pz	-10.52	< .001	Pz	-5.66	< .001	Pz	-5.81	< .001
	CPP4	-9.94	< .001	CPP4	-4.74	< .001	CPP4	-5.81	< .001
	TPP8h	-9.26	< .001	TPP8h	-5.49	< .001	TPP8h	-7.80	< .001
occipital	O1	-11.41	< .001	O1	-7.26	< .001	O1	-4.63	< .001
	Oz	-11.46	< .001	Oz	-7.79	< .001	Oz	-4.81	< .001
	O2	-10.34	< .001	O2	-7.39	< .001	O2	-4.44	< .001
low math ability, simple									
frontal	Fp1			Fp1			Fp1		
	FPz			FPz		< .001	FPz		
	Fp2			Fp2			Fp2		
	AFF7h			AFF7h			AFF7h	-3.63	.002
	AFF3			AFF3			AFF3		
	Fz	4.21	< .001	Fz			Fz		
	AFF4			AFF4			AFF4		
	AFF8h			AFF8h			AFF8h		
temporal	T7			T7			T7		
central	FCC3			FCC3	-4.43	< .001	FCC3	-6.74	< .001
	Cz			Cz	-4.02	.001	Cz	-4.50	< .001
	FCC4			FCC4			FCC4	-3.98	.001
temporal	T8			T8			T8	-4.33	< .001
parietal	TPP7h			TPP7h	-5.12	< .001	TPP7h	-8.54	< .001
	CPP3			CPP3	-5.79	< .001	CPP3	-10.66	< .001
	Pz			Pz	-4.75	< .001	Pz	-7.26	< .001
	CPP4			CPP4			CPP4	-6.09	< .001
	TPP8h			TPP8h	-3.95	.001	TPP8h	-5.77	< .001
occipital	O1			O1			O1	-5.07	< .001
	Oz			Oz	-4.30	< .001	Oz	-5.01	< .001
	O2			O2			O2	-5.97	< .001
low math ability, complex									
frontal	Fp1	-6.81	< .001	Fp1	-12.28	< .001	Fp1	-15.22	< .001
	FPz			FPz	-12.15	< .001	FPz	-15.56	< .001
	Fp2	-7.33	< .001	Fp2	-10.45	< .001	Fp2	-15.61	< .001
	AFF7h	-5.74	< .001	AFF7h	-10.02	< .001	AFF7h	-15.06	< .001
	AFF3	-13.81	< .001	AFF3	-10.21	< .001	AFF3	-16.31	< .001
	Fz			Fz	-9.89	< .001	Fz	-13.96	< .001
	AFF4	-10.22	< .001	AFF4	-10.36	< .001	AFF4	-18.05	< .001
	AFF8h	-5.35	< .001	AFF8h	-10.97	< .001	AFF8h	-18.75	< .001
temporal	T7	-10.13	< .001	T7	-11.58	< .001	T7	-17.85	< .001
central	FCC3	-13.35	< .001	FCC3	-13.12	< .001	FCC3	-18.58	< .001
	Cz	-8.33	< .001	Cz	-14.11	< .001	Cz	-24.24	< .001
	FCC4	-15.35	< .001	FCC4	-12.76	< .001	FCC4	-23.08	< .001
temporal	T8	-12.84	< .001	T8	-7.99	< .001	T8	-18.72	< .001
parietal	TPP7h	-12.16	< .001	TPP7h	-17.48	< .001	TPP7h	-23.84	< .001
	CPP3	-11.71	< .001	CPP3	-16.37	< .001	CPP3	-25.99	< .001
	Pz	-12.94	< .001	Pz	-15.28	< .001	Pz	-22.84	< .001
	CPP4	-13.74	< .001	CPP4	-15.22	< .001	CPP4	-23.18	< .001
	TPP8h	-12.04	< .001	TPP8h	-11.41	< .001	TPP8h	-22.19	< .001
occipital	O1	-10.08	< .001	O1	-13.89	< .001	O1	-16.32	< .001
	Oz	-13.07	< .001	Oz	-12.79	< .001	Oz	-15.87	< .001
	O2	-8.82	< .001	O2	-10.66	< .001	O2	-18.29	< .001
low math ability, complex vs. simple									
frontal	Fp1	-6.23	< .001	Fp1	-4.54	< .001	Fp1	-6.39	< .001
	FPz	-6.25	< .001	FPz	-6.93	< .001	FPz	-7.93	< .001
	Fp2	-8.00	< .001	Fp2	-6.59	< .001	Fp2	-6.81	< .001
	AFF7h	-7.17	< .001	AFF7h	-5.61	< .001	AFF7h	-7.49	< .001
	AFF3	-9.70	< .001	AFF3	-9.87	< .001	AFF3	-7.99	< .001
	Fz	-8.59	< .001	Fz	-8.45	< .001	Fz	-9.53	< .001
	AFF4	-7.52	< .001	AFF4	-10.43	< .001	AFF4	-8.27	< .001
	AFF8h	-6.64	< .001	AFF8h	-8.89	< .001	AFF8h	-7.87	< .001
temporal	T7	-12.34	< .001	T7	-9.21	< .001	T7	-5.90	< .001
central	FCC3	-12.16	< .001	FCC3	-6.07	< .001	FCC3	-6.50	< .001
	Cz	-8.98	< .001	Cz	-7.66	< .001	Cz	-6.39	< .001
	FCC4	-12.70	< .001	FCC4	-5.51	< .001	FCC4	-6.02	< .001
temporal	T8	-8.47	< .001	T8	-6.79	< .001	T8	-6.88	< .001

parietal	TPP7h	-6.62	< .001	TPP7h	-7.90	< .001	TPP7h	-7.25	< .001
	CPP3	-9.56	< .001	CPP3	-5.88	< .001	CPP3	-6.13	< .001
	Pz	-11.06	< .001	Pz	-5.63	< .001	Pz	-5.71	< .001
	CPP4	-10.49	< .001	CPP4	-5.30	< .001	CPP4	-5.72	< .001
	TPP8h	-6.41	< .001	TPP8h	-7.94	< .001	TPP8h	-5.90	< .001
occipital	O1	-4.16	< .001	O1	-7.25	< .001	O1	-5.51	< .001
	Oz	-8.11	< .001	Oz	-7.59	< .001	Oz	-5.92	< .001
	O2	-5.23	< .001	O2	-6.63	< .001	O2	-5.43	< .001

Note that only values for significant electrode positions ($p < .05$, Bonferroni corrected) are given. Positive values indicate event-related synchronization (ERS) and negative values indicate event-related desynchronization (ERD).

Table S5: Theta, lower and upper alpha ERS/ERD during division in individuals with high and low math ability.

site	theta	*t*	*p*	lower alpha	*t*	*p*	upper alpha	*t*	*p*
high math ability, simple									
frontal	Fp1	4.09	.001	Fp1			Fp1		
	FPz			FPz			FPz	-3.96	.001
	Fp2			Fp2			Fp2		
	AFF7h	5.61	< .001	AFF7h			AFF7h		
	AFF3			AFF3			AFF3		
	Fz			Fz			Fz		
	AFF4			AFF4			AFF4		
	AFF8h	5.47	< .001	AFF8h			AFF8h		
temporal	T7			T7			T7		
central	FCC3			FCC3			FCC3	-3.80	.002
	Cz			Cz			Cz		
	FCC4			FCC4			FCC4		
temporal	T8			T8			T8		
parietal	TPP7h			TPP7h			TPP7h	-6.31	< .001
	CPP3			CPP3	-4.05	.001	CPP3	-8.64	< .001
	Pz			Pz	-4.99	< .001	Pz	-8.59	< .001
	CPP4			CPP4	-5.18	< .001	CPP4	-5.42	< .001
	TPP8h			TPP8h	-4.96	< .001	TPP8h	-5.42	< .001
occipital	O1			O1	-4.33	< .001	O1	-6.38	< .001
	Oz			Oz	-4.89	< .001	Oz	-6.29	< .001
	O2			O2	-5.51	< .001	O2	-7.04	< .001
high math ability, complex									
frontal	Fp1			Fp1	-6.12	< .001	Fp1	-4.42	< .001
	FPz			FPz	-8.12	< .001	FPz	-8.98	< .001
	Fp2			Fp2	-5.28	< .001	Fp2	-4.02	.001
	AFF7h			AFF7h	-6.90	< .001	AFF7h	-7.90	< .001
	AFF3	-4.29	< .001	AFF3	-13.14	< .001	AFF3	-13.29	< .001
	Fz	-5.22	< .001	Fz	-12.75	< .001	Fz	-14.82	< .001
	AFF4			AFF4	-9.50	< .001	AFF4	-9.29	< .001
	AFF8h			AFF8h	-5.97	< .001	AFF8h	-6.15	< .001
temporal	T7	-7.33	< .001	T7	-17.17	< .001	T7	-15.89	< .001
central	FCC3	-13.13	< .001	FCC3	-15.35	< .001	FCC3	-15.89	< .001
	Cz	-10.08	< .001	Cz	-14.61	< .001	Cz	-15.57	< .001
	FCC4	-9.66	< .001	FCC4	-12.36	< .001	FCC4	-14.75	< .001
temporal	T8	-7.83	< .001	T8	-12.75	< .001	T8	-14.95	< .001
parietal	TPP7h	-9.02	< .001	TPP7h	-16.83	< .001	TPP7h	-19.74	< .001
	CPP3	-10.33	< .001	CPP3	-17.07	< .001	CPP3	-22.77	< .001
	Pz	-10.35	< .001	Pz	-18.26	< .001	Pz	-25.65	< .001
	CPP4	-10.83	< .001	CPP4	-17.46	< .001	CPP4	-21.55	< .001
	TPP8h	-9.25	< .001	TPP8h	-15.63	< .001	TPP8h	-19.29	< .001
occipital	O1	-7.82	< .001	O1	-15.38	< .001	O1	-16.54	< .001
	Oz	-8.49	< .001	Oz	-15.53	< .001	Oz	-16.31	< .001
	O2	-7.27	< .001	O2	-13.63	< .001	O2	-15.51	< .001
high math ability, complex vs. simple									
frontal	Fp1	-6.30	< .001	Fp1	-7.32	< .001	Fp1	-8.71	< .001
	FPz	-5.83	< .001	FPz	-8.70	< .001	FPz	-7.44	< .001
	Fp2	-5.12	< .001	Fp2	-5.44	< .001	Fp2	-6.00	< .001

	AFF7h	-4.10	< .001	AFF7h	-6.24	< .001	AFF7h	-6.21	< .001
	AFF3	-8.01	< .001	AFF3	-8.60	< .001	AFF3	-6.85	< .001
	Fz	-8.77	< .001	Fz	-8.64	< .001	Fz	-10.47	< .001
	AFF4	-10.25	< .001	AFF4	-8.10	< .001	AFF4	-9.02	< .001
	AFF8h	-4.43	< .001	AFF8h	-7.84	< .001	AFF8h	-8.92	< .001
temporal	T7	-10.22	< .001	T7	-7.50	< .001	T7	-6.34	< .001
central	FCC3	-9.24	< .001	FCC3	-6.78	< .001	FCC3	-5.65	< .001
	Cz	-8.88	< .001	Cz	-5.93	< .001	Cz	-6.19	< .001
	FCC4	-9.21	< .001	FCC4	-5.75	< .001	FCC4	-5.65	< .001
temporal	T8	-8.92	< .001	T8	-5.45	< .001	T8	-6.93	< .001
parietal	TPP7h	-10.83	< .001	TPP7h	-6.10	< .001	TPP7h	-6.65	< .001
	CPP3	-10.54	< .001	CPP3	-5.54	< .001	CPP3	-6.60	< .001
	Pz	-10.52	< .001	Pz	-5.66	< .001	Pz	-5.81	< .001
	CPP4	-9.94	< .001	CPP4	-4.74	< .001	CPP4	-5.81	< .001
	TPP8h	-9.26	< .001	TPP8h	-5.49	< .001	TPP8h	-7.80	< .001
occipital	O1	-11.41	< .001	O1	-7.26	< .001	O1	-4.63	< .001
	Oz	-11.46	< .001	Oz	-7.79	< .001	Oz	-4.81	< .001
	O2	-10.34	< .001	O2	-7.39	< .001	O2	-4.44	< .001
low math ability, simple									
frontal	Fp1			Fp1			Fp1		
	FPz			FPz			FPz		
	Fp2			Fp2			Fp2		
	AFF7h			AFF7h			AFF7h		
	AFF3			AFF3			AFF3		
	Fz			Fz			Fz		
	AFF4			AFF4			AFF4		
	AFF8h			AFF8h			AFF8h		
temporal	T7			T7			T7		
central	FCC3			FCC3			FCC3	-6.51	< .001
	Cz			Cz			Cz	-4.74	< .001
	FCC4			FCC4			FCC4	-5.98	
temporal	T8			T8			T8	-3.62	.002
parietal	TPP7h			TPP7h	-5.05	< .001	TPP7h	-7.20	< .001
	CPP3			CPP3	-5.26	< .001	CPP3	-8.81	< .001
	Pz			Pz	-4.30	< .001	Pz	-7.39	< .001
	CPP4			CPP4	-4.51	< .001	CPP4	-6.72	< .001
	TPP8h			TPP8h			TPP8h	-5.36	< .001
occipital	O1			O1			O1	-5.17	< .001
	Oz			Oz			Oz	-4.95	< .001
	O2			O2			O2	-5.52	< .001
low math ability, complex									
frontal	Fp1			Fp1	-9.57	< .001	Fp1	-11.29	< .001
	FPz			FPz	-11.51	< .001	FPz	-13.80	< .001
	Fp2			Fp2	-6.59	< .001	Fp2	-10.99	< .001
	AFF7h			AFF7h	-5.65	< .001	AFF7h	-9.10	< .001
	AFF3	-5.17	< .001	AFF3	-10.59	< .001	AFF3	-15.26	< .001
	Fz			Fz	-10.93	< .001	Fz	-17.00	< .001
	AFF4	-4.18	< .001	AFF4	-9.90	< .001	AFF4	-14.75	< .001
	AFF8h			AFF8h	-7.27	< .001	AFF8h	-10.12	< .001
temporal	T7	-8.98	< .001	T7	-11.61	< .001	T7	-16.54	< .001
central	FCC3	-8.77	< .001	FCC3	-13.50	< .001	FCC3	-20.27	< .001
	Cz	-8.08	< .001	Cz	-13.02	< .001	Cz	-22.43	< .001
	FCC4	-11.64	< .001	FCC4	-10.20	< .001	FCC4	-20.56	< .001
temporal	T8	-9.08	< .001	T8	-6.95	< .001	T8	-16.42	< .001
parietal	TPP7h	-12.18	< .001	TPP7h	-16.71	< .001	TPP7h	-21.43	< .001
	CPP3	-9.73	< .001	CPP3	-15.09	< .001	CPP3	-27.26	< .001
	Pz	-10.33	< .001	Pz	-13.90	< .001	Pz	-22.34	< .001
	CPP4	-10.90	< .001	CPP4	-13.86	< .001	CPP4	-21.25	< .001
	TPP8h	-11.39	< .001	TPP8h	-8.47	< .001	TPP8h	-17.81	< .001
occipital	O1	-7.28	< .001	O1	-12.90	< .001	O1	-16.60	< .001
	Oz	-9.86	< .001	Oz	-10.33	< .001	Oz	-14.15	< .001
	O2	-6.88	< .001	O2	-10.15	< .001	O2	-17.28	< .001
low math ability, complex vs. simple									
frontal	Fp1	-3.76	.002	Fp1	-6.22	< .001	Fp1	-5.95	< .001

	FPz			FPz			FPz		
	Fp2	-3.92	.001	Fp2	-6.67	< .001	Fp2	-4.94	< .001
	AFF7h	-6.35	< .001	AFF7h	-6.92	< .001	AFF7h	-5.54	< .001
	AFF3	-8.52	< .001	AFF3	-10.42	< .001	AFF3	-5.79	< .001
	Fz	-4.75	< .001	Fz	-8.12	< .001	Fz	-7.67	< .001
	AFF4	-7.79	< .001	AFF4	-8.95	< .001	AFF4	-8.46	< .001
	AFF8h	-5.92	< .001	AFF8h	-8.47	< .001	AFF8h	-6.76	< .001
temporal	T7	-15.90	< .001	T7	-8.05	< .001	T7	-8.37	< .001
central	FCC3	-10.49	< .001	FCC3	-9.05	< .001	FCC3	-7.37	< .001
	Cz	-7.46	< .001	Cz	-5.54	< .001	Cz	-6.56	< .001
	FCC4	-8.32	< .001	FCC4	-8.08	< .001	FCC4	-8.68	< .001
temporal	T8	-8.61	< .001	T8	-4.72	< .001	T8	-8.05	< .001
parietal	TPP7h	-13.43	< .001	TPP7h	-8.06	< .001	TPP7h	-7.48	< .001
	CPP3	-9.42	< .001	CPP3	-5.84	< .001	CPP3	-5.51	< .001
	Pz	-11.10	< .001	Pz	-4.87	< .001	Pz	-5.52	< .001
	CPP4	-12.71	< .001	CPP4	-4.88	< .001	CPP4	-6.74	< .001
	TPP8h	-11.67	< .001	TPP8h	-3.82	.002	TPP8h	-6.63	< .001
occipital	O1	-7.72	< .001	O1	-7.07	< .001	O1	-4.19	< .001
	Oz	-9.04	< .001	Oz	-6.96	< .001	Oz	-4.69	< .001
	O2	-9.21	< .001	O2	-5.85	< .001	O2	-4.46	< .001

Note that only values for significant electrode positions ($p < .05$, Bonferroni corrected) are given. Positive values indicate event-related synchronization (ERS) and negative values indicate event-related desynchronization (ERD).
